# Genetic characterization of the *Entamoeba moshkovskii* population based on different potential genetic markers

**DOI:** 10.1017/S003118202400026X

**Published:** 2024-04

**Authors:** Sanjib K. Sardar, Ajanta Ghosal, Tapas Haldar, Akash Prasad, Sweety Mal, Yumiko Saito-Nakano, Seiki Kobayashi, Shanta Dutta, Tomoyoshi Nozaki, Sandipan Ganguly

**Affiliations:** 1 Division of Parasitology, ICMR-National Institute of Cholera and Enteric Diseases (ICMR-NICED), Kolkata, India; 2 Department of Parasitology, National Institute of Infectious Diseases, 1-23-1 Toyama, Shinjuku-ku, Tokyo 162-8640, Japan; 3 Division of Bacteriology, ICMR-National Institute of Cholera and Enteric Diseases (ICMR-NICED), Kolkata, India; 4 Department of Biomedical Chemistry, School of International Health, Graduate School of Medicine, The University of Tokyo, Tokyo, Japan

**Keywords:** amoebapore C, chitinase, *Entamoeba moshkovskii*, KERP1, linkage disequilibrium, multilocus sequence typing

## Abstract

*Entamoeba moshkovskii*, according to recent studies, appears to exert a more significant impact on diarrhoeal infections than previously believed. The efficient identification and genetic characterization of *E. moshkovskii* isolates from endemic areas worldwide are crucial for understanding the impact of parasite genomes on amoebic infections. In this study, we employed a multilocus sequence typing system to characterize *E. moshkovskii* isolates, with the aim of assessing the role of genetic variation in the pathogenic potential of *E. moshkovskii*. We incorporated 3 potential genetic markers: KERP1, a protein rich in lysine and glutamic acid; amoebapore C (apc) and chitinase. Sequencing was attempted for all target loci in 68 positive *E. moshkovskii* samples, and successfully sequenced a total of 33 samples for all 3 loci. The analysis revealed 17 distinct genotypes, labelled M1–M17, across the tested samples when combining all loci. Notably, genotype M1 demonstrated a statistically significant association with diarrhoeal incidence within *E. moshkovskii* infection (*P* = 0.0394). This suggests that M1 may represent a pathogenic strain with the highest potential for causing diarrhoeal symptoms. Additionally, we have identified a few single-nucleotide polymorphisms in the studied loci that can be utilized as genetic markers for recognizing the most potentially pathogenic *E. moshkovskii* isolates. In our genetic diversity study, the apc locus demonstrated the highest *H*_d_ value and *π* value, indicating its pivotal role in reflecting the evolutionary history and adaptation of the *E. moshkovskii* population. Furthermore, analyses of linkage disequilibrium and recombination within the *E. moshkovskii* population suggested that the apc locus could play a crucial role in determining the virulence of *E. moshkovskii*.

## Graphical abstract

**Figure:**
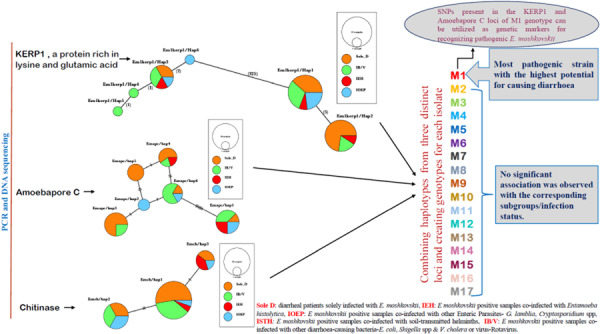

## Introduction

Amoebic infection is a complex issue as several species are morphologically indistinguishable from each other, including *Entamoeba histolytica*, *Entamoeba dispar*, *Entamoeba bangladeshi* and *Entamoeba moshkovskii* (Fotedar *et al*., 2007). This makes it challenging to accurately estimate the prevalence of each species and its potential to cause disease in humans. The cysts of a non-pathogenic amoeba, *Entamoeba hartmanni*, can also be mistaken with the pathogenic *E. histolytica* under a microscope (Burrows, 1959) adds an uncertainty. While *E. histolytica* is known to cause pathogenicity in amoebic infections, the actual prevalence of this species is likely overestimated due to these morphological overlaps. Recent research has indicated that *E. moshkovskii* might have a more significant impact on human infections than previously believed. This species has been detected in multiple countries, including the United States, Italy, Iran, Turkey, Indonesia, Colombia, Bangladesh, India, Kenya, Australia, Malaysia, Tanzania, Tunisia and Brazil (Ali *et al*., 2003; Fotedar *et al*., 2007; Khairnar and Parija, 2007; Ayed *et al*., 2008; Beck *et al*., 2008; Delialioglu *et al*., 2008; Anuar *et al*., 2012; Ngui *et al*., 2012; Shimokawa *et al*., 2012; Fonseca *et al*., 2016; Al-Areeqi *et al*., 2017; Kyany'a *et al*., 2019). In addition, *E. moshkovskii* has been identified in farm animals such as pigs, showcasing its potential for zoonotic transmission (Sardar *et al*., 2022). Moreover, it has been documented in non-human primates as well (Levecke *et al*., 2010). A study conducted in eastern India by Sardar *et al*. (2023*a*, 2023*b*) also revealed that *E. moshkovskii* is one of the causative agents of diarrhoeal incidents in humans. The research found that many patients suffering from diarrhoea, infected with *E. moshkovskii*, tested negative for other common enteric pathogens such as bacteria and viruses (Sardar *et al*., 2023*a*, 2023*b*). These findings, combined with various studies conducted in different regions, suggest the potential pathogenicity of *E. moshkovskii* in humans. Therefore, diagnosing diarrhoeal patients should include consideration of *E. moshkovskii* as a potential pathogen to ensure accurate identification of the causative agent. Neglecting this can result in undetermined cases of diarrhoeal illness, leading to improper drug treatments for patients.

Although, *E. moshkovskii* has demonstrated pathogenicity, not all genotypes within the species are linked to diarrhoeal incidence, similar to *E. histolytica*, where only 10% of infections exhibit pathogenicity (Sardar *et al*., 2023*a*, 2023*b*). Hence, it is essential to conduct genotyping of *E. moshkovskii* to accurately identify its pathogenic genotypes. In our earlier study, we have identified some significant single-nucleotide polymorphisms (SNPs) that were linked to clinical outcomes (Sardar *et al*., 2023*a*, 2023*b*). However, it is important to note that the correlation between SNPs and clinical features does not necessarily mean that they directly impact pathogenicity (Sardar *et al*., 2023*a*, 2023*b*). While genotyping using 18S rRNA is essential in phylogenetic analysis, it does not directly affect pathogenicity. Therefore, we need to explore alternative approaches to understand how *E. moshkovskii* genotypes control pathogenicity.

Efficient identification and genetic characterization of clinical isolates from endemic areas worldwide play a crucial role in understanding the impact of parasite genomes on amoebic infections. Multilocus sequence typing (MLST) is a valuable tool widely used in various studies, offering a convenient and reproducible system for typing (Urwin and Maiden, 2003; Klint *et al*., 2007; Bom *et al*., 2011; Xia and Xiong, 2014; de Vries *et al*., 2015). Several MLST systems have been developed successfully for characterizing the strains of *E. histolytica* (Sardar *et al*., 2023*b*). The selection of appropriate genetic markers is vital for genotype analysis. In our MLST study, we have integrated 3 potential genetic markers associated with the incidence of diarrhoeal *E. moshkovskii* infection. These markers include KERP1, a protein rich in lysine and glutamic acid, amoebapore C (apc) and chitinase. By incorporating these markers into our genotyping system, we aim to enhance the characterization of *E. moshkovskii* strains and gain further insights into their role in diarrhoeal infections.

KERP1 is a protein abundant in lysine and glutamic acid. This protein is found on the exterior of the *E. histolytica* parasite in the form of a trimeric protein complex. KERP1 is a significant factor associated with the virulence of *E. histolytica* and possesses unique characteristics that differentiate it from other known proteins. This unique protein comprises 25% lysine and 19% glutamic acid residues. Its initial discovery was prompted by its interaction with the brush border of human enterocytes (Perdomo *et al*., 2013). KERP1 localizes at the trophozoite plasma membrane and exhibits close association with intracellular vesicles (Seigneur *et al*., 2005). Analyses of gene expression indicate elevated KERP1 transcript levels in virulent strains, while non-virulent *E. histolytica* strains display lower protein levels (Santi-Rocca *et al*., 2008). *In vivo* investigations employing a hamster model of amoebic liver infection further supported the significance of KERP1 as a virulence factor (Santi-Rocca *et al*., 2008, Perdomo *et al*., 2013). Using antisense methods to decrease KERP1 expression stopped liver abscess formation, highlighting the significance of the protein in amoebic pathogenicity (Baxt and Singh, 2008). While the precise function of KERP1 during infection remains unclear, it is undoubtedly engaged in trophozoite interactions, promoting host cell death, phagocytosis and initiating inflammation in ALA (amoebic liver abscess) development (Nozaki and Bhattacharya, 2014). Therefore, KERP1 is considered a crucial virulence factor for *Entamoeba*. The KERP1 gene from *E. moshkovskii* displays homology with the corresponding gene in *E. histolytica*. The kerp1 genes of *E. histolytica* (EHI_098210), *Entamoeba nuttalli* (ENU1_189420) and *E. moshkovskii* (EMO_099600) exhibit noteworthy similarities. Specifically, there is a 100% self-match in *E. histolytica*, a 97% amino acid identity across the entire protein in *E. nuttalli* and a 45% amino acid identity over a portion of the protein in *E. moshkovskii* (Weedall, 2020).

apc is another protein implicated in the virulence of *E. histolytica*. Earlier research has unveiled the presence of important SNPs in the upstream region of the apc protein gene within *E. histolytica* (Bhattacharya *et al*., 2005). These SNPs exhibit a notable connection with the disease outcomes of amoebiasis (Bhattacharya *et al*., 2005). Although the fact that the precise role of apc remains somewhat subtle, its influence on the severity of the disease is becoming clearer through these genetic associations. This gene exhibits homologous counterparts in various other *Entamoeba* species, including the *E. dispar* strain SAW760 (EDI_206610), the *Entamoeba invadens* strain IP1 (EIN_133650) and *E. moshkovskii* Laredo (EMO_119370) (Das *et al*., 2021). Given its association with disease severity, apc presents itself as a promising candidate for inclusion in our genotyping investigation.

The third gene analysed in our MLST study is the chitinase of *E. moshkovskii*. Within the *Entamoeba* species, there are multiple chitinases that share a conserved type 18 glycohydrolase domain (de la Vega *et al.*, 1997). The process of amoebic encystation involves the expression of chitinase (de la Vega *et al*., 1997). These chitinase genes contain repetitive DNA sequences that display notable variations among the isolates. Specifically, the repeat types and arrangement patterns within *E. histolytica* show a considerable inter-isolate diversity. While the involvement of chitinase (EC 3.2.1.14) in cyst wall formation is plausible, its role remains unverified. Chitinase functions by breaking down chitinase, a polymer made up of *N*-acetyl-d-glucosamine units joined by *β*-1,4 linkages. Although there is a suggestion that *Entamoeba* Chitinase contributes to cyst wall modification during encystation, supporting evidence is limited (Mi-Ichi *et al*., 2021). In our MLST study, we have included a potential genetic marker: a Chitinase gene from *E. moshkovskii* (EMO_056190), which shares sequence similarity with *E. histolytica* (KM1_098160).

The objective of MLST analysis was to identify genotypes that exhibit a statistical correlation with the co-infection status of *E. moshkovskii*, drawing insights from our epidemiological dataset. We conducted comparative genetic assessments of distinct *E. moshkovskii* populations within diverse co-infection subgroups. We also intend to uncover genetic markers, such as SNPs, that display significant connections with the occurrence of diarrhoeal incidence attributed to *E. moshkovskii* infections. It is essential to investigate the correlation between *E. moshkovskii* genotypes and infection status to acquire a better understanding of the molecular mechanisms that play a role in *E. moshkovskii* pathogenesis. Furthermore, it is important to gather genome information of infecting strains from endemic areas worldwide to expand our understanding of this relationship. This study aimed to identify specific genetic variations associated with *E. moshkovskii* infection. Furthermore, the research aimed to explore the impact of genetic variations within *E. moshkovskii* subgroups on their infection outcomes, specifically concerning diarrhoeal incidence. This includes cases of sole *E. moshkovskii* infection and those with co-infection involving other enteric pathogens, using an MLST approach.

## Materials and methods

### Samples

The study utilizes the 68 samples that tested positive for *E. moshkovskii*. These specific samples had been identified using microscopy and polymerase chain reaction (PCR) techniques targeting the 18S rRNA locus, as described in a prior publication (Sardar *et al*., 2023*a*, 2023*b*). This study involved the characterization of 5 subgroups within the *E. moshkovskii* populations: Sole D, IOEP, IEH, ISTH and IB/V. Sole D represents patients experiencing diarrhoea solely due to *E. moshkovskii* infection. IEH signifies *E. moshkovskii*-positive samples co-infected with *E. histolytica.* IOEP denotes *E. moshkovskii*-positive samples co-infected with other enteric parasites, including *Giardia lamblia* and *Cryptosporidium* spp. ISTH corresponds to *E. moshkovskii*-positive samples co-infected with soil-transmitted helminths. Lastly, IB/V includes *E. moshkovskii*-positive samples co-infected with other diarrhoeal agents, such as *Escherichia coli*, *Shigella* spp., *Vibrio cholera* or the Rotavirus. For a comprehensive understanding of the study area, stool sample collection, DNA extraction process, detection of *E. moshkovskii* in the specimens and data collection of co-infection status, we recommend referring to the methodology section of our earlier study by Sardar *et al*. (2023*a*, 2023*b*).

### PCR amplification

Positive samples for *E. moshkovskii* were chosen to amplify 3 target genes: KERP1, apc and chitinase. The amplification process was carried out using a reaction mixture with a volume of 50 μL. This mixture included 5 units of TaKaRa Ex-Taq polymerase, PCR buffer at a 1× concentration, 0.2 μm of both forward and reverse primers and 3 μL of stool DNA samples with a concentration of 50 ng μL^−1^. The amplification reactions were carried out using a thermal cycler PCR system from Applied Biosystems (Foster City, USA). The PCR cycling procedure commenced with an initial denaturation phase at 94°C, lasting for 5 min. This was followed by 35 amplification cycles, each comprised of distinct steps. These cycles consisted of a denaturation step at 94°C for a duration of 30 s, an annealing phase at 56°C (for KERP1) for 25 s, a polymerization step at 72°C lasting for 45 s and a concluding extension stage at 72°C, maintained for 7 min. The amplification process for apc and chitinase followed a similar pattern, except that the annealing temperature was set at 57°C and the polymerization time was reduced to 35 s.

Primer sequences, expected PCR product sizes and annealing temperatures employed are provided in Table 1. Following amplification, the PCR products were subjected to electrophoresis on agarose gel (Seakem^®^ LE Agarose, Lonza) and subsequently visualized under a UV transilluminator following staining with 0.5 μm mL^−1^ ethidium bromide.

**Table 1.** Primer sequences, expected PCR product sizes and annealing temperatures of the targeted loci

Name of the primer	Primer sequence (5′–3′)	Annealing temperature (°C)	Product size (bp)
Emkerp1_F	TATGAGCGTTGGGGAGATTC	56	594
Emkerp2_R	CTTCCCGCCATCAAAAATAA		
Emapc_F	TCTTGAAAGTCTTTGCGCCA	57	449
Emapc_R	TCCTCCTCTCGTAGTCCAAA		
EmChitinase_F	TGTGGTGTTTCAAAAGTTTCCA	57	357
EmChitinase_R	CAACACAAAAATAAATAGTCATTCACG		

### DNA sequencing

PCR products of the expected sizes were extracted using a Roche Gel Extraction Kit following the manufacturer's protocols. Their yield was subsequently verified through gel electrophoresis. The purified PCR products were then subjected to direct sequencing using the corresponding amplification primers in both forward and reverse directions. This sequencing process employed the BigDye Terminator v3.1 Cycle Sequencing Kit from Applied Biosystems, USA. The obtained sequences were analysed using an ABI3730 sequencer.

### Sequence analysis

The obtained DNA sequences were aligned using the clustalW multiple sequence alignment program from GenomeNet Bioinformatics resources and edited manually. Subsequently, the DNA sequences from the 3 loci were combined to determine a genotype. Alphanumerical codes were then assigned to the obtained genotypes. We also identified the SNPs present in the obtained local isolates. All the sequences were aligned with reference sequences obtained from AmoebaDB using the MultAlin online tool and thereafter the SNPs were identified. The representative nucleotide sequences of each haplotype reported in this study have been deposited in NCBI GenBank. We also inferred relationships between the haplotypes by constructing a minimal-spanning haplotype network using Pop-ART v1.7. To elucidate the connections within the sequence data, we employed a colour-coded representation of isolates based on their co-infection status.

### Statistical analysis

Categorical data analysis was conducted using GraphPad Prism 9, CA, USA. The association between the genotypes/repeat patterns and clinical phenotypes was assessed using the Fisher's exact test. Statistical significance was defined as a *P* value <0.05 in all instances.

## Results

### Successful amplification of the target loci

Out of the total 68 samples, successful amplification was achieved in 33 samples across 3 designated target loci (Supplementary file 1). Nevertheless, the remaining samples did not yield successful amplification for all 3 loci due to the presence of low DNA concentration in the stool samples. Some samples exhibited faint amplification, which was inadequate for sequencing, while others displayed no distinct bands upon agarose gel electrophoresis. The lack of amplification observed in certain samples could be attributed to either a low cyst concentration of *E. moshkovskii* in fecal samples or issues with the quality of DNA. Furthermore, the existence of genetic polymorphisms at the specified target sites could also contribute to the observed lack of amplification. Although implementing a nested PCR technique has the potential to enhance the amplification rate, we did not prefer to adopt this approach to prevent the risk of cross-contamination. The representative haplotype sequences obtained in this study have been submitted to NCBI GenBank under accession numbers OR621050–OR621064.

### Single-nucleotide polymorphisms

A total of 22 SNPs and 3 deletions were detected within the 3 genes.

We successfully obtained complete gene sequences for the kerp1 and chitinase genes. In contrast, our analysis of the apc gene involved a partial sequence, encompassing both a partial coding region and a 322 bp long intron region, as predicted in AmoebaDB.

**Figure 1.**
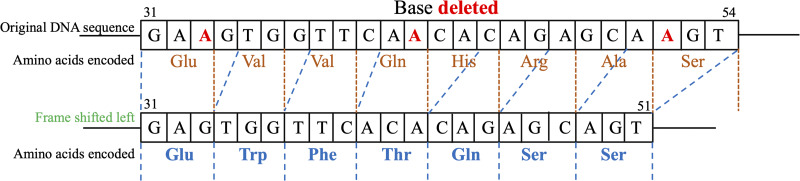
Illustration of the frameshift mutation identified in the kerp1 locus of *Entamoeba moshkovskii* in eastern India. Deletion of 3 adenine (A) bases occurred at positions 33, 42 and 52 in the original DNA sequence. As a result, the amino acid sequence –Glu–Val–Val–Gln–His–Arg–Ala–Ser– was replaced with –Glu–Trp–Phe–Thr–Gln–Ser–Ser–, leading to the loss of 1 amino acid in the altered sequence.

In Kerp1, we have identified a total of 9 SNPs along with 3 specific deletions denoted as 33A, 42A and 52A. These deletions resulted in the removal of a sequence of 3 base pairs, consequently altering the corresponding amino acid sequence. Specifically, the sequence –Glu–Val–Val–Gln–His–Arg–Ala–Ser– was substituted with –Glu–Trp–Phe–Thr–Gln–Ser–Ser– (Fig. 1). As a consequence of the deletion, 1 amino acid was lost in the replaced sequence. Interestingly, despite this alteration in the amino acid sequence, our analysis did not reveal any statistically significant associations between this deleted amino acid stretch and various co-infected groups. Furthermore, we have identified 5 SNPs labelled 23A/C, 25A/C, 26C/T, 27T/G and 28G/A. These variants have shown a significant correlation with diarrhoeal incidences in cases of sole infection with *E. moshkovskii*, with the corresponding *P* values of 0.0261 for each SNP. Conversely, the remaining 3 SNPs did not exhibit any statistically significant connections with the co-infected groups.

In the apc gene, a total of 10 SNPs were identified. Among these, 9 SNPs were located within the intronic region of the gene. Four intronic SNPs exhibited a statistically significant correlation with the occurrence of sole diarrhoea, which we refer to as the Sole D group (as shown in Table 6). Within these 4 SNPs, 3 – namely 420T/A (*P* = 0.0224), 564T/A (*P* = 0.2916) and 523A/T (*P* = 0.0538) – demonstrated a positive statistical correlation with the incidence of sole diarrhoea. Additionally, 1 SNP, 299A/T, displayed a negative association (*P* = 0.0315) with sole diarrhoeal incidence. These intronic SNPs may have the capacity to decrease protein levels by potentially influencing splicing processes (Wang and Sadee, 2016). Moreover, the identified SNPs can also serve as genetic markers. It is important to note that the intron region reported in this study was based on the predicted genomic sequence of the apc gene (EMO_119370) sourced from AmoebaDB.

The chitinase locus analysis revealed the presence of 3 SNPs, all of which lacked statistical significance when correlated with the specific subgroups of *E. moshkovskii.* Details of these identified SNPs within the target loci of the studied isolates are given in Table 2.

**Table 2.** Identified SNPs within the target loci of the studied isolates

Target loci	Base pair (bp) analysed	SNP^a^ position including deletion	Amino acid substitution	Co-infection status			
				Sole D	IEH	IOEP	IB/V
Kerp1	546	Deletion 33A, 42A, 52A	12–17 VVQHRA/WFT|QS	X^c^	X^c^	X^c^	X^c^
		23A/C	8Q/P	*P*^b^ = 0.0261 *χ*^2^ = 4.90	X^c^	X^c^	X^c^
		25A/C	9T/L	*P*^b^ = 0.0261 *χ*^2^ = 4.90	X^c^	X^c^	X^c^
		26C/T	9T/L	*P*^b^ = 0.0261 *χ*^2^ = 4.90	X^c^	X^c^	X^c^
		27T/G	11T/L	*P*^b^ = 0.0261 *χ*^2^ = 4.90	X^c^	X^c^	X^c^
		28G/A	10V/I	*P*^b^ = 0.0261 *χ*^2^ = 4.90	X^c^	X^c^	X^c^
		374A/G	125E/G	X^c^	X^c^	X^c^	X^c^
		405G/T	135N/K	X^c^	X^c^	X^c^	X^c^
		432C/G	144D/E	X^c^	X^c^	X^c^	X^c^
		441T/A	147D/E	X^c^	X^c^	X^c^	X^c^
apc	407	299A/T	Intron region	*P*^b^ = 0.0315 *χ*^2^ = 4.626 (negative association)	X^c^	X^c^	X^c^
		325A/T	Intron region	X^c^	X^c^	X^c^	X^c^
		327C/T	Intron region	X^c^	X^c^	X^c^	X^c^
		360T/A	Intron region	X^c^	X^c^	X^c^	X^c^
		370T/A	Intron region	X^c^	X^c^	X^c^	X^c^
		398A/C	Intron region	X^c^	X^c^	X^c^	X^c^
		420T/A	Intron region	*P*^b^ = 0.0224 *χ*^2^ = 5.215	X^c^	X^c^	X^c^
		564T/A	Intron region	*P*^b^ = 0.2916 *χ*^2^ = 1.112	X^c^	X^c^	X^c^
		523A/T	Intron region	*P*^b^ = 0.0538 *χ*^2^ = 3.718	X^c^	X^c^	X^c^
		607A/T	55K/N	X^c^	X^c^	X^c^	X^c^
Chitinase	306	75T/C	25Y/Y (synonymous)	X^c^	X^c^	X^c^	X^c^
		223A/T	75I/L	X^c^	X^c^	X^c^	X^c^
		286T/C	96S/P	X^c^	*P*^b^ = 0.0001 *χ*^2^ = 17.22 (negative association)	X^c^	X^c^

a The positions of all SNPs were reported with reference to first base of the start codon, which is designated as position 1.

b Probability value of the particular association.

c Does not have any association with disease outcomes.

*χ*^2^ = chi-square value.

In the obtained DNA sequences of the 3 loci, a majority of the SNPs were characterized as non-synonymous mutations.

### Association between genotype and co-infection status

After conducting sequencing on all the samples, we identified a total of 6 distinct haplotypes in the Kerp1 group. Notably, one of these haplotypes, Emk3, displayed a complete 100% match with the reference sequence EMO_099600. In contrast, the apc group also exhibited 6 distinct haplotypes, all of which differed from the reference sequence EMO_119370. Within the chitinase gene sequences, we observed 3 distinct haplotypes. Notably, one of these haplotypes, Emch1, exhibited similarity to the reference sequence EMO_056190. These individual haplotypes were subsequently pooled together to construct the respective genotypes.

After combining 3 distinct loci, this study has successfully identified 17 distinct genotypes labelled M1 through M17. Among these genotypes, 6, specifically M1, M3, M5, M8, M9 and M17, were identified in multiple isolates (Table 3). Notably, genotype M1 exhibited a statistically significant association with the sole diarrhoeal group within *E. moshkovskii* infection, as indicated by a *P* value of *P* = 0.0394. While M9 and M17 were found in Sole D and IOEP subgroups with multiple occurrences, their presence did not show any statistically significant associations with their respective groups. The M12 genotype was detected in several groups, except for the Sole D group. The remaining genotypes were found in both the Sole D group and other co-infected groups. This research demonstrated that the M1 genotype holds the highest potential for being a pathogenic strain of *E. moshkovskii*.

**Table 3.** List of identified genotypes (M1–M17) resulting from the combination of 3 independent studied loci

Co-infection status	Sample ID	Sequence pattern			Genotype
		Kerp1	apc	Chitinase	
Sole D	EM_IND/1	Emk2	Emapc3	Emch1	M1^a^
	EM_IND/3	Emk1	Emapc5	Emch1	M2
	EM_IND/4	Emk2	Emapc3	Emch1	M1^a^
	EM_IND/5	Emk2	Emapc3	Emch1	M1^a^
	EM_ID/6	Emk1	Emapc6	Emch2	M3
	EM_IND/10	Emk1	Emapc4	Emch1	M4
	EM_IND/11	Emk3	Emapc4	Emch1	M5^b^
	EM_IND/12	Emk2	Emapc3	Emch3	M6
	EM_IND/15	Emk1	Emapc5	Emch2	M7
	EM_IND/16	Emk2	Emapc3	Emch1	M1^a^
	EM_IND/17	Emk1	Emapc1	Emch3	M8
	EM_IND/19	Emk3	Emapc4	Emch2	M5^b^
	EM_IND/21	Emk2	Emapc5	Emch1	M9^b^
	EM_IND/22	Emk2	Emapc3	Emch1	M1^a^
	EM_IND/26	Emk2	Emapc5	Emch1	M9^b^
IB/V	EM_IND/34	Emk1	Emapc6	Emch2	M3^b^
	EM_IND/36	Emk2	Emapc3	Emch1	M1^a^
	EM_IND/37	Emk3	Emapc4	Emch2	M5^b^
	EM_IND/39	Emk1	Emapc3	Emch1	M10
	EM_IND/40	Emk4	Emapc6	Emch1	M11
	EM_IND/47	Emk1	Emapc1	Emch1	M12^a^
	EM_IND/48	Emk3	Emapc1	Emch1	M13
	EM_IND/49	Emk1	Emapc1	Emch1	M12^a^
	EM_IND/50	Emk5	Emapc6	Emch1	M14
	EM_IND/51	Emk2	Emapc6	Emch2	M15
IEH	EM_IND/57	Emk1	Emapc1	Emch3	M8^b^
	EM_IND/60	Emk2	Emapc1	Emch3	M16
	EM_IND/61	Emk3	Emapc4	Emch1	M5^b^
IOEP	EM_IND/63	Emk3	Emapc2	Emch2	M17^b^
	EM_IND/64	Emk1	Emapc1	Emch3	M8^a^
	EM_IND/65	Emk1	Emapc1	Emch1	M12^a^
	EM_IND/67	Emk6	Emapc2	Emch2	M17^b^
	EM_IND/68	Emk1	Emapc6	Emch2	M3^b^

a Statistically associated with the Sole D group, *P* = 0.0394 (*χ*^2^ = 4.244).

b Multiple occurrences but not statistically significant.

### Genetic diversity

The haplotype diversity (*H*_d_) across individual polymorphic loci ranges from 0.572 to 0.833. Within the 33 samples of *E. moshkovskii*, the number of haplotypes ranges from 2 to 6. Among the 3 examined loci, the apc locus showed the highest *H*_d_ value of 0.833 and the highest number of haplotypes (6). Additionally, the apc locus exhibits the highest observed nucleotide diversity. Moderate levels of haplotype diversity were revealed within the kerp1 and chitinase loci, with the *H*_d_ values of 0.572 and 0.589, respectively (Table 4). Additionally, these loci exhibited moderate levels of nucleotide diversity (*π*), with kerp1 exhibiting 0.0048 and *chitinase* exhibiting 0.0035. Among the examined loci, the apc locus showed the greatest number of polymorphic sites (10), while the chitinase locus showed the fewest (3). Tajima's *D* statistics revealed positive values for all loci. These positive values could imply the presence of either a population bottleneck or balancing selection. The presence of a substantial variety of genotypes within this group, coupled with the positive Tajima's *D* value, may support the hypothesis of balancing selection (Table 4). However, given that these values did not show statistical significance, confirming these results would require a larger sample size.

**Table 4.** Different genetic diversity indices of *Entamoeba moshkovskii* population based on 3 target loci

	Haplotype details		Nucleotide details		Number of segregating sites (S)	Tajima's *D*	LD (|*D*′|)	*R*_m_
	Number of haplotypes	*H*_d_	*K*	*π*				
Kerp1	5	0.572	2.59	0.0048	9	0.51	*Y* = 1.0000 + 0.000*X*	0
apc	6	0.833	3.97	0.0097	10	1.90	*Y* = 0.9779 − 0.1659*X*	1
Chitinase	3	0.589	1.08	0.0035	3	1.06	*Y* = 1.0000 + 0.000*X*	0

Assessment of intragenic recombination revealed that apc exhibited 1 recombination event (*R*_m_s), and LD was incomplete in that locus, whereas the remaining 2 markers (with complete LD) did not have *R*_m_s.

The combined nucleotide sequences of the 3 target loci were either 1259 bp (due to 3 deletions in Kerp1) or 1256 bp long and included 22 variable sites. The *H*_d_ value was 0.93. Tajima's *D* statistics for the concatenated sequences was 1.42, supporting the idea of balancing selection (Table 5). To validate these findings further, a larger sample size would be needed.

**Table 5.** Different genetic diversity indices of *E. moshkovskii* population based on using concatenated multilocus sequences

	Haplotype details		Nucleotide details					
	Number of haplotypes	*H*_d_	*K*	*Π*	Number of segregating sites (S)	Tajima's *D*	LD (|*D*′|)	*R*_m_
Kerp1 + apc + chitinase	17	0.93	7.65	0.0060	22	1.42	*Y* = 0.8169 + 0.1059*X*	1

### Linkage disequilibrium (LD) and recombination analyses of target loci

We assessed intragenic LD and the count of potential recombination events for each target locus. At the apc locus, an incomplete intragenic LD value was observed (|*D*′|*Y* = 0.9779 − 0.1659*X*), with *Y* representing the LD value and *X* indicating the nucleotide distance in kilobases. The incomplete intragenic LD value at the apc locus suggests a non-random distribution of its alleles within the *E. moshkovskii* population. Conversely, complete LD values (|*D*′|*Y* = 1.0000 − 0.0000*X*) were discovered at the Kerp1 and chitinase loci, indicating a random distribution of alleles for these 2 genes. The analysis of intragenic recombination revealed a single potential event (*R*_m_) exclusively at the apc locus. Conversely, no recombination events were observed at the Kerp1 and chitinase loci, signifying that the alleles of these 2 genes are distributed randomly in the studied population (Table 4).

Our study isolates were analysed for an overall interlocus LD and the number of potential recombination events. The concatenated multilocus sequence data were used for this purpose. The analysis revealed an incomplete LD value (|*D*′|*Y* = 0.8169 + 0.1059*X*) with a single potential recombination event in the population. This was observed when the concatenated sequences of all 3 loci were analysed. An incomplete interlocus LD value (|*D*′|*Y* = 0.7682 + 0.4469*X*) was discovered between chitinase and Kerp1, indicating a single recombination event. However, upon further analysis of concatenated sequences among apc + chitinase and apc + Kerp1, 2 potential recombination events with incomplete interlocus LD values were observed (Table 6). This interesting finding suggests a possible non-random association of the apc locus with both chitinase and Kerp1.

**Table 6.** Different genetic diversity indices, interlocus LD and recombination analyses of *E. moshkovskii* population using concatenated multilocus sequences

	Haplotype details		Nucleotide details					
	Number of haplotypes	*H*_d_	*K*	*π*	Number of segregating sites (S)	Tajima's *D*	LD (|*D*′|)	*R*_m_
Including apc								
apc + chitinase	11	0.90	3.97	0.0071	13	1.87	*Y* = 0.9708 − 0.4183*X*	2
apc + Kerp1	13	0.89	6.56	0.0069	19	1.37	*Y* = 1.067 − 0.5529*X*	2
Excluding apc								
Chitinase + Kerp1	9	0.83	3.67	0.0043	12	0.776	*Y* = 0.7682 + 0.4469*X*	1

### Interlocus LD and recombination analyses of *E. moshkovskii* population from different co-infection/Sole infection groups

We conducted interlocus LD and recombination analyses on the *E. moshkovskii* population from 4 groups (Sole D, IB/V, IEH and IOEP) using concatenated multilocus sequence data. Our analysis revealed that only the apc locus showed a single recombination event in intralocus LD analysis. To further analyse interlocus LD values, we focused on concatenated multilocus sequences both including and excluding the apc locus. Interestingly, we found that the inclusion of the apc locus increased recombination events. However, LD analysis of concatenated multilocus sequences from the IEH and IOEP groups produced a complete interlocus LD value (|*D*′|*Y* = 1.0000 + 0.0000*X*) with no recombination events in either case. Based on the complete interlocus LD value (|*D*′|*Y* = 1.0000 + 0.0000*X*) and the absence of recombination events in the IEH and IOEP groups, it appears that these groups might be isolated compared to the others. However, since most concatenated sequences show at least 1 recombination event, inter-population genetic recombination may occur among the different subpopulations (Tables 7 and 8). One notable finding from this study is that the IEH and IOEP populations of *E. moshkovskii* are undergoing a speciation process due to their isolation.

**Table 7.** Interlocus LD and recombination investigations within the *E. moshkovskii* population across various co-infection/sole infection groups, utilizing combined multilocus sequences excluding apc loci

	Populations	No. of samples	No. of polymorphic sites analysed	No. of pairwise comparisons	No. of significant pairwise comparisons (Fisher's exact test)	LD (|*D*′|)	*R*_m_
Excluding apc	All	33	12	66	12	*Y* = 0.7682 − 0.4469*X*	1
	Sole D + IB/V	25	10	45	11	*Y* = 0.7121 − 0.5057*X*	1
	Sole D + IEH	18	8	28	11	*Y* = 0.7423 − 0.8854*X*	1
	Sole D + IOEP	20	10	45	21	*Y* = 0.8196 − 0.4000*X*	1
	IB/V + IEH	13	10	45	11	*Y* = 0.6397 − 0.4699*X*	1
	IB/V + IOEP	15	12	66	11	*Y* = 0.8170 − 0.2243*X*	1
	IEH + IOEP	8	10	45	1	*Y* = 1.0000 − 0.0000*X*	0

**Table 8.** Interlocus LD and recombination investigations within the *E. moshkovskii* population across various co-infection/sole infection groups, utilizing combined multilocus sequences after inclusion of apc loci

	Populations	No. of samples	No. of polymorphic sites analysed	No. of pairwise comparisons	No. of significant pairwise comparisons (Fisher's exact test)	LD (|*D*′|)	*R*_m_
Including apc	All	33	22	231	51	*Y* = 0.8169 + 0.0398*X*	3
	Sole D + IB/V	25	20	190	40	*Y* = 0.8280 + 0.0717*X*	3
	Sole D + IEH	18	18	153	46	*Y* = 0.9681 − 0.6813*X*	3
	Sole D + IOEP	20	20	190	60	*Y* = 0.8645 + 0.0426*X*	3
	IB/V + IEH	13	20	190	28	*Y* = 0.8253 − 0.1884*X*	2
	IB/V + IOEP	15	22	231	38	*Y* = 0.8806 + 0.1126*X*	2
	IEH + IOEP	8	19	171	16	*Y* = 1.0000 − 0.0000*X*	0

A clear observation from the analyses of both intragenic and interlocus LD was that genetic recombination predominantly took place at the apc locus.

### Haplotype network construction

**Figure 2.**
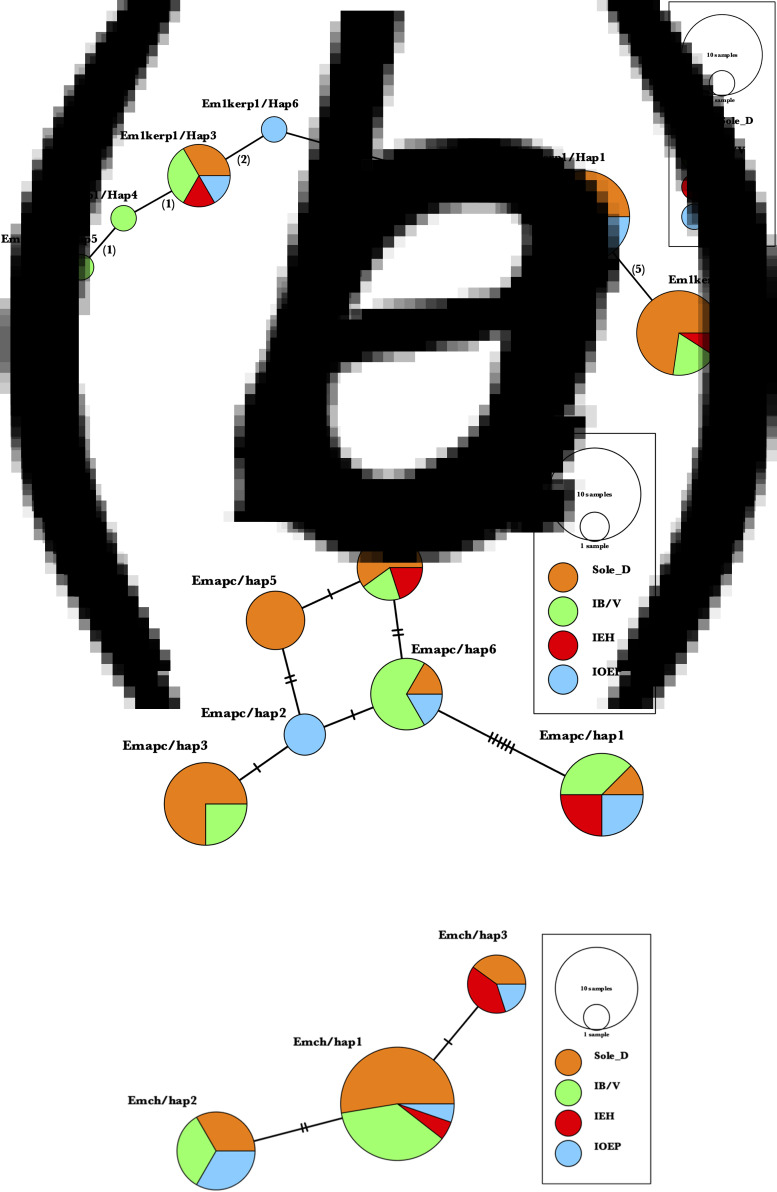
Minimal spanning haplotype network of *E. moshkovskii* haplotypes obtained from individuals with different co-infected subgroups. The network illustrates the genetic relationships between the haplotypes, with each circle representing a unique haplotype and the size of the circle indicating its frequency. The colours of the circles correspond to the co-infection status. (a) Lysine and glutamic acid-rich protein 1 (KERP1); (b) apc (pore-forming peptides) and (c) chitinase.

Haplotype grouping was conducted using the KERP1, apc and chitinase markers. The inclusion of various co-infected subgroups in the haplotype network did not result in any apparent impact on isolate grouping (Fig. 2a–c). In Kerp1, Em1kerp1/Hap1 has emerged as the predominant haplotype within all subgroups and is likely the ancestral haplotype. However, in our analysis, the Em1kerp1/Hap3 genotype is reported as the original DNA code (prototype) for the Kerp1 protein. This determination is based on its perfect alignment with the reference sequence EMO_099600 from AmoebaDB, as well as its significantly high prevalence across all co-infected subgroups, supporting its prototype status. A frameshift was observed due to the deletion of 3 bases, leading to the origin of other mutant types from Em1kerp1/Hap3. The remarkable adaptability of Em1kerp1/Hap3, as indicated by its prevalence across all subgroups, further underscores its prototype status. However, it was observed that Em1kerp1/Hap6 originated from either Em1kerp1/Hap1 or Em1kerp1/Hap3 and was specifically found in the IOEP subgroups. The Em1kerp1/Hap2 was at 1 mutational step away from Em1kerp1/Hap1 and was not observed in the IOEP subgroup. The Em1kerp1/Hap6 haplotype forms a branch with 3 descendant haplotypes, namely Em1kerp1/Hap3, Em1kerp1/Hap4 and Em1kerp1/Hap5.

In apc, 6 haplotypes (Emapc/hap1 to Emapc/hap6) have been identified. The most frequent haplotype, Emapc/hap1, is likely the ancestral haplotype and is predominant in all 4 subgroups. Emapc/hap1 is 6 mutational steps away from Emapc/hap6. However, Emapc/hap2 and Emapc/hap4 are 1 and 2 mutational steps away, respectively, from Emapc/hap6. Emapc/hap5 has emerged as a descendant of both Emapc/hap2 and Emapc/hap4 haplotypes. Interestingly, Emapc/hap5 is exclusively observed in parasites from the Sole D group. In chitinase, we detected 3 distinct haplotypes: Emch/hap1, Emch/hap2 and Emch/hap3. Among these, Emch/hap1 was the most prevalent and presumed to be the ancestral haplotype. Emch/hap2 and Emch/hap3 were found to be at 2 and 1 mutational step away from Emch/hap1, respectively. Notably, Emch/hap2 was not observed within the IEH subgroups.

## Discussion

Our goal was to analyse the genetic makeup of various isolates of *E. moshkovskii* and its association with virulence factors found in *E. histolytica*. We focused specifically on the loci of lysine and glutamic acid-rich protein 1 (KERP1), apc (pore-forming peptides) and chitinase. KERP1 is found on the trophozoite plasma membrane and internal vesicles, where it plays a crucial role in establishing amoeba–cell contacts and the development of liver abscesses (Santi-Rocca *et al*., 2008; Perdomo *et al*., 2013). Amoebapore forms ion channels or pores in lipid membranes, depolarizing target cells (Leippe *et al*., 1991). The expression of amoebapores is necessary for the complete manifestation of virulence in *E. histolytica*, particularly in the context of amoebic liver abscesses (Zhang *et al*., 2004). Chitinase, on the contrary, breaks down chitinase, a *β*-1,4-linked polymer of *N*-acetyl-d-glucosamine, and is believed to be involved in re-modelling the cyst wall during encystation in *Entamoeba* (Chatterjee *et al*., 2009; Mi-Ichi *et al*., 2021). In our study, we employed PCR amplification using specific primers designed for targeting these genetic loci. This approach has the potential to provide novel insights into the co-infection dynamics of *E. moshkovskii*.

Accurate identification and genetic characterization of clinical isolates from endemic regions worldwide provides a valuable tool for understanding the impact of parasite genome on the outcomes of amoebic infections. Earlier research has established that tRNA-linked short tandem repeat (STR) loci serve as surrogate markers for determining disease outcomes (Ali *et al*., 2012). In our current study, we have genetically characterized *E. moshkovskii* populations with varying co-infected groups using the above-mentioned coding genes. The Kerp1 gene exhibited the highest number of SNPs, with 5 of them being associated with diarrhoea incidence and potentially serving as genetic markers. These SNPs may also play a role in modulating the pathogenicity of *E. moshkovskii*. The Apc gene displayed a number of significant SNPs; the exact impact of which remains to be determined. However, the presence of these SNPs can serve as a genetic marker for diarrhoeal diseases caused by isolates similar to those observed in Kerp1. Most of the SNPs observed in our study were non-synonymous, which aligns with earlier findings for *Mycobacterium tuberculosis*, where only 36 out of 101 identified SNPs were synonymous (Baker *et al*., 2004). This trend was also noted by Das *et al*. (2021) in their MLST study of *E. histolytica* (Das *et al*., 2021). Interestingly, the chitinase gene showed only synonymous SNPs and was found to be the most conserved gene among the studied loci, with only 3 SNPs observed. In contrast, the chitinase of *E. histolytica* is highly polymorphic and contains STR units, whereas the chitinase of *E. moshkovskii* is not a highly polymorphic gene as observed in this study (Das *et al*., 2014). The reported SNPs could potentially play a role in drug sensitivity in *E. moshkovskii*, similar to how certain SNPs have been linked to multidrug resistance in *Plasmodium falciparum*, as reported by Coulibaly *et al*. (2022).

Recombination events were only identified at the apc locus. The presence of co-infection-specific SNPs, potential recombination events within the apc locus and various non-synonymous base changes all suggest that this region of the genome is under selection pressure. As such, these observations may indicate that apc could play a crucial role in determining the virulence of *E. moshkovskii*. The precise function of apc in *E. moshkovskii* pathogenesis remains unknown. While investigating another gene, Kerp1, we identified a significant number of SNPs, despite the absence of detected potential recombination events. Therefore, further studies with a larger sample size are required to gain a better understanding of the role of Kerp1 in the population structure of *E. moshkovskii*. It is important to note that this is the first molecular epidemiology-based study to date conducted on the role of different genes in the pathogenicity of *E. moshkovskii*.

The findings of this study suggest a correlation between the parasite genotypes and *E. moshkovskii* infection status. This study is the first to explore the direct link between parasite factors and the infection dynamics of *E. moshkovskii*. However, further biomarkers are necessary to comprehensively understand the role of the parasite genome.

## Conclusion

The recent research has unveiled the genetic composition of *E. moshkovskii* isolates under investigation, establishing their link to infection dynamics. Through the analysis, numerous significant SNPs within specific genetic regions have been detected. These SNPs exhibit the potential to influence, either directly or indirectly, the pathogenicity and drug sensitivity of *E. moshkovskii*. The investigation has also pinpointed distinct clusters of isolates that display genetic segregation. Moreover, the study supports the hypothesis that a connection exists between parasite genotypes and infection dynamics.

## Supporting information

Sardar et al. supplementary material Sardar et al. supplementary material

## Data Availability

Representative sequences obtained in this study were deposited in GenBank under the accession numbers OR621050–OR621064.

## Supplementary material

The supplementary material for this article can be found at https://doi.org/10.1017/S003118202400026X.
