# Supplementary material for: Genetic characterization of the Entamoeba moshkovskii population based on different potential genetic markers
Source: Parasitology. 2024 Mar 11;151(4):429–39. doi: 10.1017/S003118202400026X (PMC11044060; doi:10.1017/S003118202400026X)
Supplement: Sardar et al. supplementary material [file S003118202400026Xsup001.pdf]

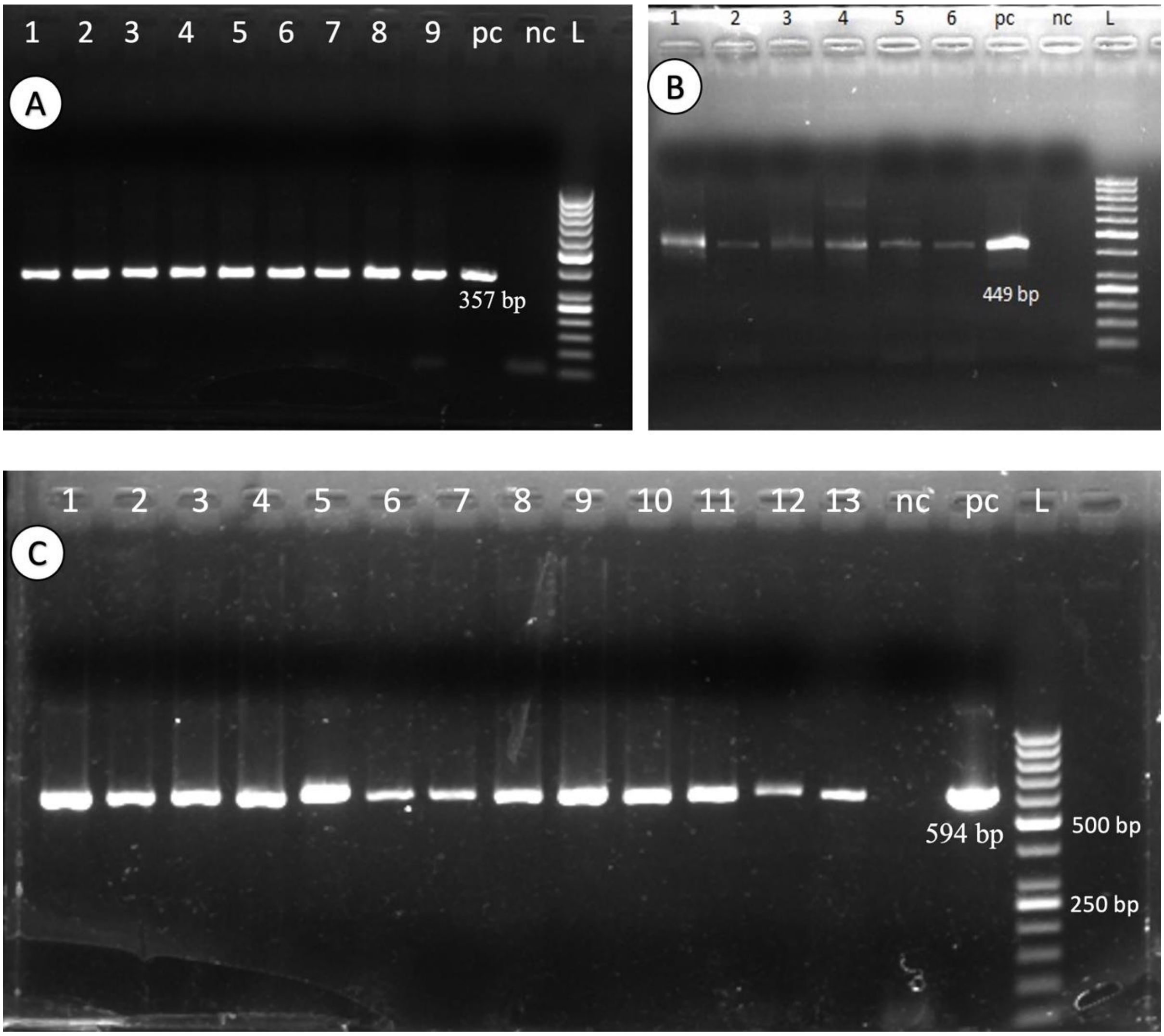

Supplementary Figure 1 Amplification of different target loci of *E. moshkovskii.* **A.** Chitinase **B.** Amoebapore C **C.** Kerp1. **Nc:** Negative control, **Pc:** Positive control**, L:** 50bp ladder (Fermentas).
